# The Negro, from a Medical Standpoint

**Published:** 1886-08

**Authors:** 


### *THE NEGRO, FROM A MEDICAL STANDPOINT.*

Dr. P. Tipton, of Selma, Ala., in a carefully-written article published in the *Sanitarian*, gives some valuable facts regarding the negro race in the South. He first shows that their death-rate is over thirty per 1,000, more than double that of their white-skinned rivals. The death-rate among the Blacks exceeds the birth-rate. The women usually have some uterine disease, which lessens their productiveness. Among the slaves, phthisis was unknown, but now the disease is four times more frequent among the Blacks than the Whites. Phthisis is now the greatest foe of the negro race. Malarial diseases are rare, and when they do occur, are of a very mild type. One-half the male population is syphilitic, but the disease is mild and usually runs its course without treatment. Cancer of the womb is rare; lacerated cervix common. Deafness, insanity, diphtheria and croup are also rare, while hysteria, rheumatism and alcoholism are common. Unless the sanitary condition of the race is soon improved, it is threatened with extinction.

